# Contraception to women’s economic empowerment: A narrative review^☆^

**DOI:** 10.1016/j.worlddev.2025.107167

**Published:** 2025-12

**Authors:** Jocelyn E. Finlay, Mariam Gulaid, Chiseche Mibenge, Nyovani Madise, Naa Dodua Dodoo, John Stover, Michelle Weinberger, Michelle O’Brien, Marita Zimmermann

**Affiliations:** a Harvard TH Chan School of Public Health, 665 Huntington Avenue, Boston 02115, USA; b Guttmacher Institute, USA; c African Institute for Development Policy, Malawi; d Avenir Health, USA; e Institute for Disease Modeling, Gates Foundation, USA

**Keywords:** Economics, Demography, Contraception, Fertility, Economic activity, Empowerment, Gender

## Abstract

The pathway linking contraceptive access and use to women’s economic empowerment is explored in this narrative review. Contraception can have a direct effect on women’s economic empowerment through supporting bodily autonomy, but the main way in which the pathway links is through fertility. Contraception impacts the timing, spacing, and number of births, and these elements of fertility each impact the process of women’s economic empowerment. These pathways are explored with a focus on sub-Saharan Africa. With access to contraception, girls and women can plan their reproductive lives and this enables them to plan their education and economic lives. With access to contraception, girls are more likely to complete school and go on to higher-paying jobs and livelihoods. Contraception enables women to plan their birth intervals and manage the joint roles of childrearing and labor force participation. Contraception enables women to realize their desired number of children and make their own choice regarding their time use in work and child-rearing. Investing in adolescents brings the highest long-term gains to women’s economic empowerment. Policies that dismantle gender-discriminating social norms will bolster the positive effects of contraception on women’s economic empowerment. While the context of the evidence reviewed here shaped the interpretation of results and how these successes are adapted to other environs, this narrative review contributes to a growing compendium of evidence that suggests that investing in programs that support women in their access and use of contraception is an effective way to support women in their economic empowerment process.

## Highlights

• A compendium of peer-review literature shows that, with sensitivity to context, contraceptive access and use can have a positive impact on women’s economic empowerment.
• Contraception is a way for women to plan their reproductive lives, and this enables effective planning of their education and economic lives.
• Planning of the timing, spacing and limiting of births is enabled by contraception, and this planning paves the way for women’s economic empowerment.
• Discriminatory gender norms can hamper the empowerment process for women.

## 1 Introduction

Supporting the pathway to women’s economic empowerment in sub-Saharan Africa (SSA) has wide reaching benefits (Nabimanya, Mayanja, & Kyarikunda, 2024), and investments in family planning (in this paper, specifically contraception) have been shown to be an effective mode (Joshi and Schultz, 2007, Miller, 2010) to provide this support. Having control of her reproductive life enables a woman to plan, and have control over, her economic life (Gammage et al., 2020, Goldin and Katz, 2002). Investing in contraception as a pathway to women’s empowerment is an established policy recommendation (Joshi and Schultz, 2013, Miller, 2010) with strong supporting evidence within high-income country (HIC) settings (Bailey, 2006, Bailey, 2013, Bailey et al., 2012, Goldin and Katz, 2002). In low- and middle-income countries (LMICs), much of the seminal research has been focused in the South Asian region where a deeper understanding of the empowerment process has been established (Kabeer, 1999, Kabeer, 2005, Kabeer, 2015), and where robust evidence of the positive impacts of contraception on dimensions of empowerment has been found (Joshi & Schultz, 2007). While some studies have shown low levels of empowerment amongst women in SSA (Ewerling et al., 2020, Miedema et al., 2018),^1^ similar to the low levels in South Asia, there is a lack of literature on the empowerment process in the African region. In this narrative review, we focus on the economic empowerment process in SSA and examine how contraception impacts that process. To do this, we draw on the existing body of literature, with specific consideration of the role that the context of each study played in shaping and interpreting the results.

The far-reaching impact of investments in contraception is illustrated by seminal studies in Matlab (Joshi and Schultz, 2007, Joshi and Schultz, 2013) and Navrongo (Debpuur, Phillips, Jackson, Nazzar, & Binka, 2002). In their 2012 summary article, Canning and Schultz (2012) review these seminal studies to illustrate how contraceptive access and use contributed to the achievement of the Millenium Development Goals (MDGs) of eradicating extreme poverty and hunger (MDG1), achieving universal primary education (MDG2), and promoting gender equality and empowering women (MDG3) (Canning & Schultz, 2012). We are now in the era of the Sustainable Development Goals (SDGs), and the thesis set out by Canning and Schultz (2012) can be extended to illustrate the broader impacts of reproductive health improvements (of which contraceptive access and use is a subset) on poverty (SDG 1), hunger (SDG 2), health (SDG 3), education (SDG 4) and decent work for all (including addressing gender wage gaps and the informal labor market) (SDG 8) (Esquivel & Sweetman, 2016). Empowerment of women and girls is SDG 5. The targets within SDG 5 include measuring decision-making over sexual relations, contraceptive use, and reproductive health care, which addresses the need for reproductive empowerment within the broader development framework. Targets also include measures of women’s equal rights to, and control over, economic resources. Within the SDG framework, reproductive and economic empowerment are intertwined. The examination of the existing literature on the impact of contraceptive access and use on women’s economic empowerment informs the understanding, measurement and advancement of the SDGs.

A collection of review papers (Canning and Schultz, 2012, Finlay and Lee, 2018, Finlay, 2021, Gammage et al., 2020) have shown evidence of the connection between reproductive empowerment^2^ and economic empowerment. The connection between reproductive empowerment and economic empowerment (Gammage et al., 2020) highlights that development policy that focuses solely on educating girls and getting women into the labor force (as in Brown (2021)) will not materialize benefits unless attention is paid to women’s reproductive health and rights (Gammage et al., 2020). In this narrative review, we highlight the mechanisms by which this connection manifests, with a focus on contraceptive access and use to women’s economic empowerment.

While there is a direct effect of contraceptive access and use on women’s economic empowerment, the main pathway is realized through the elements of fertility – timing, spacing and limiting (Finlay, 2021). The mechanisms that play out along these fertility pathways differ across contexts. For example, in LMICs, politics have shifted reproductive health agendas towards birth spacing and away from teen childbearing (May, 2017), and limiting fertility has been interpreted as Western coercion and population control (May, 2017). Furthermore, as female labor force participation is a dimension of (although not synonymous to) economic empowerment the dominance of the informal labor market in LMICs can mean that policies such as maternity leave, which aim to help women balance competing demands of childcare and formal employment, have little traction (Finlay, 2021). Finlay (2021) shows that the connection between fertility and women’s labor force participation is context specific by demonstrating that the mechanism connecting the variables of interest (fertility and women’s labor force participation) varied according to a country’s average income levels. Finlay (2021) showed that the literature illustrated that low-, middle- and high-income countries faced different trajectories of how fertility connected to work, and this had significant implications for the development of policies that aimed to promote decent work for women (SDG 8). Accordingly, in this paper, we also consider contextual variables as modifiers of the conceptual pathways linking contraception to women’s economic empowerment.

Women’s economic empowerment is important for the development of society as a whole, as it advances human development in low- and middle-income countries (Balasubramanian, Ibanez, Khan, & Sahoo, 2024), and advances human rights (Pradhan et al., 2023). Women’s empowerment can be bolstered by supporting women’s productivity in agriculture, promoting micro-enterprise, facilitating financial access, and encouraging formal labor force participation (Balasubramanian et al., 2024). These interventions, however, do not address the intersection of reproductive empowerment and economic empowerment that is an overarching theme in a woman’s life and development of her overall welfare (Gammage et al., 2020). Without reproductive empowerment, a woman will struggle to harness the benefits of any assistance to her economic empowerment (Gammage et al., 2020). Thus, in this narrative review, we explore the literature that connects one element of reproductive empowerment – contraceptive access and use – to women’s economic empowerment to understand the pathway between these mutually beneficial forms of empowerment.

### 1.1 Women’s economic empowerment: theory

Women’s economic empowerment is defined by elements of resources, agency and achievement (Kabeer, 1999).^3^ Resources, including assets (such as land, housing, livestock, savings) and income (monetary income and in-kind/bargained income), are needed for women to materialize their preferences.^4^ However, access to resources alone is not a sufficient condition of empowerment. Women must have agency, control and decision-making power over these resources (Kabeer, 1999). And to “achieve” empowerment, women must be able to realize their own preferences (Raj, Dey, & Lundgren, 2021).

Agency is most frequently measured in terms of decision-making agency (Annan et al., 2021, Kabeer, 1999). For choices to be considered part of the empowerment process, those choices must offer the woman transformative agency such that her welfare is elevated beyond its former trajectory (Kabeer, 1999). This transformative^5^ agency may destabilize gender-based inequalities,^6^ and it is not certain that all members of the household and community value this transformation (Kabeer, 1999). “Achievement”, within the definition of empowerment, “refers to the capacity to gain an understanding of and control over personal circumstances and social and political forces in order to achieve goals, improve living conditions, and gain mastery over one’s life” (Condren, 2024). Alternatively, achievement has recently been recognized as being defined by the individual woman, presenting a woman-centered approach, where her preferences are realized (Raj et al. 2021). The woman- centered approach provides a framework of measurable constructs that combines critical consciousness and choice, agency and backlash, and goal achievement to define the empowerment process. The approach takes account of the individual and her community, addressing internal attributes, social norms, and external contexts and resources, and how these factors improve or hinder the empowerment process (Raj et al., 2024).

The goals of women’s economic empowerment are defined at the individual level as a form of self-determination (Kabeer, 1999, Raj et al., 2021) which is “the freedom of individuals or collectives to live or act without consultation or approval from others” (Raj et al. 2021); at the household level (aiming to overcome household poverty and improve household welfare) (Buvinic, Gupta, & Casabonne, 2009); and, at the aggregate level which aims for the human right to equality and non-discrimination and more generally gender equality (Kabeer, 2015, Raj et al., 2021, Santos Silva and Klasen 2021). There is not one definition of women’s economic empowerment, nor a singular measure thereof (Buvinic et al., 2020, Laszlo et al., 2020) with the data researchers have for use in analysis to date measures of empowerment have commonly included metrics of women’s labor force participation, type of work, control of earnings, and decision-making power over household purchases. Other niche data sets are better at encompassing a truer definition of empowerment, covering the resources, agency and achievement elements, the idea that empowerment is a process, and the idea that empowerment is relative. One such dataset that caputures this more complete definition of empowerment is the Women’s Agriculture Empowerment Index (WAEI) (Alkire et al., 2013).^7^

### 1.2 Context matters

In the case of the impact of contraceptive access and use on women’s economic empowerment, if an intervention yields success, or a study shows a positive relationship, this does not imply that the same study will have the same positive impact in another context (Ali et al., 2023, Weinberger et al., 2019). Scaling up or adapting programs from one context to another must be done thoughtfully for effective policy making (Williams, 2020). In this paper we focus on the empowerment process in SSA. Though this is an extremely diverse region and the same cautions of adaptability of studies apply, for the purpose of this paper, confining the geographic context to one region enables the consideration of the role of household level contextual variables such as education (Nguyen-Phung & Nthenya, 2024) or religion (Banjo, 2024).

The context in which studies are conducted comes with implicit assumptions that shape the hypotheses (Cai & Pleskac, 2023). Initially, theories of women’s labor force participation response to fertility were developed with the high-income country setting in mind (Adda et al., 2017, Goldin and Katz, 2002). Most high-income country settings have social safety nets (for example, maternity leave (Earle, Raub, Sprague, & Heymann, 2023)), functioning healthcare institutions to ensure access to reproductive health (contraception, abortion care, ante- and post-natal care, (Forum, 2015)), and social norms that value women’s contribution to the workforce (Goldin & Katz, 2002), balancing childcare, and households which function as a team (unitary households) (Duflo & Udry, 2004). These conditions do not easily translate to the LMIC settings.

The context in which a study was conducted also shapes the interpretation of results. The literature in this review includes studies conducted in SSA that demonstrate results that oppose theory – more education for teens leaves them worse off (Baird et al., 2015, Baird et al., 2016); labor force participation increases with an extra child (Heath, 2017). These results from LMICs that oppose theory illustrate that theories created using data from HICs often include contextual assumptions that were not recognized at the time of their development.

Context also impacts the social norms that modify the conceptual pathway between contraception and women’s economic empowerment (Liu & Lapinski, 2024). For example, in an HIC context, the notion of choices (Bongaarts, 2024, Gunther and Harttgen, 2016) and deliberate trade-offs often frames women’s experience of timing and spacing childbearing and whether or not, and how many hours to, work (Goldin & Katz, 2002). Many women can choose when to have a child, how many children to have, and consider time-use of working or not, how much she works, and what type of work she does (Goldin & Katz, 2002). However, for most women in LMICs there are not the same social safety nets nor social norms to support choice (Onyina & Baye, 2024). In LMICs, women often work in the informal sector (Olu-Owolabi, Amoo, Samuel, Oyeyemi, & Adejumo, 2020), and are living in households that may not function as an (economic) collective (Duflo & Udry, 2004). There are weaker financial institutions for savings and loans to smooth consumption over the life cycle (Despard, Friedline, & Martin-West, 2020). Work may be necessary to curb poverty (Kabeer, 2015). Additionally, in many LMICs, and particularly in Africa, fertility choices are often not regulated independently by the woman. Male partners, and sometimes other relatives (e.g., mothers in law) are instrumental in fertility decision-making (Bankole and Singh, 1998, Church et al., 2023, Gudu et al., 2023). Thus, in an LMIC context, individual choice may not underpin a woman’s time trade-offs between childbearing, childcare, and labor force participation as it does in HICs.

Preferences for the number of children a woman wants may not be formed and articulated in the same way in SSA (Gunther & Harttgen, 2016) as in HICs. While across all income settings, more empowered women have preferences for fewer children (Atake & Ali, 2019), shaping these preferences and actualizing them is also a part of the empowerment process (Dadzie et al., 2024). If we take the woman- centered approach to defining and measuring empowerment (Raj et al., 2024) then an articulation of one’s own preferences is a vital step in self-determination. Acknowledging that preferences may be shaped and expressed differently by women around the world (Gunther & Harttgen, 2016) is an important ingredient in understanding how context can impact the definition and measurement of empowerment (Bedigen, 2022).

### 1.3 Women’s economic empowerment in sub-Saharan Africa

There are multiple interrelated forms of empowerment – economic, personal and socio-cultural. Recent research using data from Kenya found that women who were empowered economically were also likely to be empowered personally and socio-culturally (Nabimanya et al. 2024), but women who were empowered personally and socio-culturally were not necessarily empowered economically (Nabimanya et al. 2024). Within this Kenyan sample, only 22 percent of women were reported to be economically empowered (that is, reported to have a say in the use of her own income/earnings, the purchase of large household properties such as land or house equipment and have a say on use of her husband’s or partner’s income or earnings). In that study, it was found that women who were employed were nine times more likely to be economically empowered than women who did not work, in a sample where 54 percent of the women reported to be employed. In this sub-Saharan African context, over half of the women are employed – making them a step closer to being economically empowered compared to their unemployed counterparts. While these employed women were nine times more likely to be empowered than the unemployed counterparts, very few of the employed women had decision-making power over their earnings – the employed women had resources but not agency and thus cannot claim to be economically empowered (Nabimanya et al. 2024).

Indeed, across SSA, women’s economic empowerment rates are low (Williams, Väisänen, & Padmadas, 2022). Analysis of cross-sectional data from 33 sub-Saharan African countries found an average economic empowerment score of three out of a possible nine on the author’s scale, ranging from 1.48/9 in Niger to 4.11/9 in South Africa (Williams et al., 2022). The authors build their scale of economic empowerment as a principal components construct of labor market outcomes (employment status, income), access to resources (education, financial, land ownership), and economic decision-making (who makes decisions regarding use of the woman’s income and her husband’s income, and about household expenditure on large household items), following the methods of others who had used the same Demographic and Health Survey (DHS) data (Buvinic et al., 2020). While the DHS has many limitations in terms of measuring empowerment (e.g., the data are a repeated cross section, are not longitudinal, and the responses of decision-making questions are not available in all surveys), it has been a good resource to explore dimensions of empowerment in SSA, including Ethiopia, Gabon, Gambia, Comoros, Congo Democratic, Cote d’Ivoire, Cameroon, Chad, Kenya, Liberia, Mali, Malawi, Mozambique, Namibia, Nigeria, Sierra Leone, Tanzania, Tonga, Uganda, Zimbabwe, Zambia, Burkina Faso (Soharwardi & Ahmad, 2020). Dimensions such as women’s work status, income type (money or in-kind), participation in decision-making, self-esteem and self-confidence, can be measured using the DHS and are useful to glean an overview of empowerment in the region and across countries, as authors’ have done in Rwanda (Musonera & Heshmati, 2017).

For the remainder of this paper, we outline the methodology of this narrative review and present the results of our findings. We first present research that has shown the direct effect of contraceptive use on women’s economic empowerment. Then we provide details of the three primary pathways through which contraception impacts elements of fertility (timing, spacing, limiting) and the subsequent impact on economic empowerment, and its elements (such as education, labor force participation and decision-making power). In the discussion we address factors that modify the link from contraception to empowerment.

## 2 Methodology

To conduct this review, peer-reviewed academic publications were collected, read, analyzed, and embedded within the narrative of complementing literature examining the impact of contraception on women’s economic empowerment.

The authors began by identifying seminal papers. First, Goldin and Katz (2002). Though published decades ago for the United States of America context, Goldin and Katz illustrated influential results of the mechanism by which contraception access and use impacts women’s education and labor force effort. Secondly, Nabimanya et al (2024) provided a key starting point for gathering recent and relevant peer review articles based on SSA. Finally, existing review papers (Canning and Schultz, 2012, Finlay and Lee, 2018, Finlay, 2021, Gammage et al., 2020) were collated and the references within these review articles examined.

Using Web of Science, we were able to look backwards at the references within these seminal papers and forwards to the peer review papers that had cited them. We examined empirical research from 2002 (marked by the Goldin and Katz paper) to the present.^8^ From the seminal papers, we followed a snowball search method. Branching outwards from the seminal papers, the authors reviewed titles and abstracts to identify literature relevant to the relationships between contraception and women’s economic empowerment. Papers that were deemed relevant by the coauthors were read in detail, summarized, and synthesized in this narrative review to explore the pathway of contraceptive access and use to women’s economic empowerment.

## 3 Results

### 3.1 Direct effect of contraception on women’s economic empowerment

In an SSA context, qualitative analysis amongst a sample of women in Ethiopia found that contraceptive use resulted in a higher empowerment as contraception enabled women to have control over their bodies, reproduction and fertility (Alano & Hanson, 2018). This qualitative study highlighted the discussion of reproductive control directly resulting in greater empowerment.

In South Asia, in a recent study in India, the direct effect of contraceptive access and use on women’s (economic) empowerment has been shown to be statistically significant and has been attributed to the use of contraceptives enabling women’s mobility and elevating decision-making power (Dhak, Saggurti, & Ram, 2020). Using two-stage-least-squares analysis to overcome the reverse causality of empowerment influencing contraceptive use, the authors found that contraceptive use had a positive impact on mobility and decision-making power in India. The authors found that sterilization, and modern and traditional contraceptives had a positive impact on mobility (to market, healthcare center and outside the village) and decision-making (over seeking health care, household purchases and visiting relatives) (Dhak et al., 2020). The authors discuss the pathways by which contraception can impact empowerment outcomes and note that contraception offers a way for women to have control over their bodies, namely family size and birth spacing. They suggest that this weakens the patriarchal norms and the perception of women’s domestic role (Dhak et al., 2020), which is consistent with qualitative analysis in Ethiopia (Alano & Hanson, 2018). The authors also discuss how reproductive control enables women to seek education and work, contributing the family income and expanding their social networks – all contributors to women’s empowerment (Dhak et al., 2020).

Also in South Asia, the Matlab site in Bangladesh has been a source of longitudinal data to examine the impact of contraceptive access and use on women’s work and wages (Barham et al., 2021, Joshi and Schultz, 2007, Ruthbah, 2020). In the working paper version of their paper, Joshi and Schultz (2007) use data from treatment and control areas in Matlab, Bangladesh, where treatment households received door-to-door outreach family planning and maternal child health program services for the extended period from 1977 to 1996. Fertility rates were observed to be lower in the treatment areas. Education played an important mediating role in connecting contraceptive use to women’s empowerment. It was found that private earnings, and the return to schooling (as in return on investment), among women living in the treatment areas were three times as large per year of schooling compared to the total sample mean (Joshi & Schultz, 2007). It was also found that the better educated women in the treatment groups (i.e., those who had access to contraceptive products and services) had greater asset holdings of farmland, ponds and orchards, housing and savings/jewelry. The authors discussed that the greater asset holdings indicated that in the treatment areas, women with access to contraception had fewer children and substituted their income and wealth away from (unwanted) children to the accumulation of physical assets across the life course (Joshi & Schultz, 2007). The authors also examined the impact of contraceptive use by treatment area on child welfare and found that women who were in the treatment area had fewer children overall, reported higher levels of education in their sons (but not daughters) - a finding that is consistent with the quality-quantity tradeoff (Becker & Gregg Lewis, 1973) and son preferences (Pande & Astone, 2007).

More recently, using this same data from the Matlab site, a study concluded that the door-to-door family planning program had lifelong impacts on the women in the treatment area – increasing their contraceptive use, reducing completed fertility, and lengthening birth intervals (Barham et al., 2021). However, the authors found that there were no significant effects on economic outcomes in Matlab 35 years after the intervention. That is, there was no effect of treatment (of the family planning program) on annual wages, annual earnings, employment status, savings or productive asset ownership (such as cattle, sewing machine or loom), as shown in the appendix of that paper (Barham et al., 2021). The conflicting results from the 2007 study (Joshi & Schultz, 2007) and the 2021 study (Barham et al., 2021), highlight that time (year) is also a contextual factor that can influence the measured impact of contraceptive access and use on women's economic empowerment.

Miller (2010) estimates the positive impact of Profamilia in Colombia on girls’ education attainment. Profamilia was first introduced in 1965 and over three decades it became Colombia’s main family planning provider. Across this same time period the total fertility rate declined in Colombia at a faster rate than any other Latin American country (Miller, 2010). For their methodological approach, the author leveraged the staggered scale-up of the program to apply a natural experiment. They found that girls who aged into the Profamilia program had higher education attainment and greater formal labor force participation than earlier cohorts who did not have access to the program in their late teens. Miller (2010) concludes that family planning is one of the “best-regarded development interventions”. His study highlights the importance of the framework and pathway through which a family planning program’s success is examined; the program had a very small effect on completed fertility but a large effect on age at first birth and subsequent positive effects on education attainment and formal workforce participation.

In another study examining the direct effect of contraceptive use to women’s empowerment, the authors use longitudinal data from Kenya, Nigeria and Senegal (O’Brien, Zimmermann, Eitmann, Chao, & Proctor, 2023). Using principal components analysis to parse empowerment variables that indicate egalitarian beliefs, reproductive control and autonomy, the authors found that contraceptive use had a low – and even negative – impact on these empowerment outcomes in Kenya and Senegal, but a robust positive impact on empowerment outcomes in Nigeria. The vast variability in results (negative, low, and positive) linking contraceptive use to empowerment highlights the importance of considering geographic context in the examination of how contraceptive use impacts women’s empowerment (O’Brien et al., 2023), even within a continent, such as SSA.

While it was not an examination of empowerment outcomes *per se*, it is worth highlighting that the Navrongo study found a positive impact of contraceptive access on reproductive health knowledge, use, and fertility outcomes in a Ghanaian context (Debpuur et al., 2002). The Navrongo Community Health and Family Planning Project was a two-part program: training and mobilization of community health nurses; and the involvement of volunteer social organizations to rally support for community health and family planning services. The study was not extended to examine the impact on economic or empowerment outcomes, but the initial effort of the two-part program showed a positive direction for the women and families of Navrongo in their reproductive empowerment. Conversely, in a recent study in Burkina Faso, researchers found that improving access to contraception had little to no effect on birth rates (Dupas et al., 2025).

In this section, studies that examine the direct effect of contraception on women’s empowerment were considered. Together these studies highlight that contextual factors of continent, time or year of the study (comparing Joshi and Schultz (2007) and Barham et al. (2021) in Bangladesh, for example), cohort (Miller, 2010), and country (O’Brien et al., 2023) yield varying results that affirm or refute the positive impact contraceptive access and use has on women’s empowerment. While these studies examine the direct effect of contraception on women’s economic empowerment, the suggested pathway is, for the most part, through fertility. In the next section, we examine this pathway in greater detail. Fertility is considered as three different elements, timing of first birth (which overlaps with themes of adolescent fertility or early childbearing), birth spacing, and the number of children. Per the literature, these elements of fertility illicit distinct pathways by which contraception impacts empowerment.

### 3.2 The impact of contraception on fertility timing and women’s economic empowerment: connecting teen fertility, education, job quality and earnings

Contraception enables women to have control over timing of their first birth: adolescents can complete school, achieve higher education (Ardington, Menendez, & Mutevedzi, 2015) and pursue higher quality careers (Baird et al., 2015, Baird et al., 2016, Goldin and Katz, 2002). In a study based in ruralKwaZulu-Natal, South Africa (Ardington et al., 2015) – where teen childbearing was high even by South African averages^9^ – it was found that young mothers were 26 percent less likely to complete high school and attained 2.8 years less education than older mothers. While teen pregnancy resulted in dropping out of school for many, using longitudinal data, the authors were careful to control for selection bias and showed that women who go on to be teen mothers were not systematically worse off in terms of education prior to the birth. The authors found evidence that while some teen mothers returned to school following the birth of their child, they never fully caught up in terms of their education attainment compared to older mothers (Ardington et al., 2015).^10^

While early childbearing can cause teen mothers to drop out of school (Ardington et al., 2015), other studies consider how incentives to stay in school can reduce teen pregnancy (Duflo, Dupas, & Kremer, 2015). Both Ardington et al. (2015) in South Africa and Duflo et al. (2015) in Kenya combine their analysis of teen pregnancy with Human Immunodeficiency Virus (HIV) prevention and prevalence testing. For girls in these two sub-Saharan African contexts, the risk of unprotected sex extends beyond pregnancy and to HIV infection as well. Thus, in this context, the decision-making process around risky sexual behavior confounds pregnancy and HIV infection.

In a Kenya-based study (Duflo et al., 2015), the quality of education impacted the results of curbing teen pregnancy and the “effect of education on fertility goes beyond … the ‘incarceration effect’ of school on teen fertility” (Black et al., 2008, Duflo et al., 2015). Indeed, the interaction between teen pregnancy and education means that school is not just a “safe place” to avoid early childbearing, and that the time in school is contributing to the girls’ productive quality education.

In the US context, Goldin and Katz (2002) outlined a model using the introduction of the contraceptive pill in the 1970s to show that the sudden increase in access (and use) of reliable contraception enabled women to not only increase their years of education but also the quality of their education. That is, with access to reliable contraception and the ability to control the timing of first births, women were able to choose four-year college degrees that led to higher paying career paths (law and medicine, for example). This finding came with caveats. The authors noted that the benefits of the rise in education (years and degree type) were only possible if social norms were attuned to women’s desire for this change. Social norms must also adjust such that older ages of marriage and older age of first birth were acceptable. Social norms must be flexible to value women with a higher education. This adjustment to the social norms means that potential husbands value older, more educated, women as wives and mothers (Goldin & Katz, 2002).

It was interesting to see that when the increase in education occurred at a small scale in a Malawi-based study, and social norms did not adjust to value older more educated women, then the women who stayed in school were disadvantaged in the marriage market (Baird et al., 2015, Baird et al., 2016). This is a good example of how family planning programs cannot target interventions on a small group and expect universally positive outcomes. Small interventions can generate unintended consequences; in this case, higher education lead to lower marriage options (Baird et al., 2015, Baird et al., 2016). It is unreasonable, and unrealistic, to expect that adolescent girls can push against the tide of gender norms and social norms without societal support and an enabling environment.

Using an instrumental variables approach to isolate the causal pathway, a Madagascar study found that teen childbearing caused school discontinuation (Herrera Almanza & Sahn, 2018) and increased labor force participation, particularly in the informal labor market (Herrera, Sahn, & Villa, 2019). Another study evaluating a multifaceted policy intervention attempts to jump-start adolescent women's empowerment in Uganda by simultaneously providing them vocational training and information on sex, reproduction, and marriage. The authors found that for four years post intervention, adolescent girls in treated communities were more likely to be self-employed and less likely to be unemployed. Teen pregnancy, early entry into marriage/cohabitation, and the share of girls reporting sex against their will also fell sharply (Bandiera et al., 2020).

The pathway of contraception to women’s empowerment is shown through girls and young women having control over their reproductive lives (with contraceptive access and use) and the timing of their first (teen) births. Completing quality education (Goldin & Katz, 2002) is enabled by contraceptive access and use as women can reliably plan their time – control over their reproductive lives enables control over their current and future economic lives. However, the time gained from delaying childbearing must be used productively in quality education (Duflo et al., 2015, Goldin and Katz, 2002) and be valued within the construct of social norms (Baird et al., 2015, Baird et al., 2016).

Education leads to greater gender equality in that there is greater concordance between couples over decision-making in the household (Anderson, Reynolds, & Gugerty, 2017). For adolescents and young women, contraceptive access and use enables delayed childbearing and continued education (Ardington et al., 2015, Herrera Almanza and Sahn, 2018), better job opportunities (Bandiera et al., 2020, Herrera et al., 2019). This pathway to empowerment from contraceptive access and use begins from these teen years, and the state of empowerment is realized (thanks to education and being in a better job) within their own household later in life (Anderson et al., 2017). Thus, to study the full arch of contraceptive access and use on women’s economic empowerment for teens, the study period needs to span the teen years and into the woman’s 20 s and 30 s when she is part of managing a household.

### 3.3 Connecting contraceptive use to fertility spacing and women’s economic empowerment

The case for healthy birth spacing enabled by contraceptive use is the dominant family planning discourse across SSA (May, 2017), and it has been noted that “ ‘birth spacing’ (associated with health) has become virtually the only accepted rationale in SSA, with ‘birth limitation’ (associated with control) perceived as imperialistic and tainted by Western values.” (May, 2017). Despite the promotion of healthy birth spacing, a recent study found that 44 % of women across 35 sub-Saharan African countries have had sub-optimal birth spacing of less than 33 months (Mare et al., 2023). A field experiment in Malawi found that a post-partum family planning intervention could be effective in safeguarding healthy birth intervals (Karra, David Canning, Guo, & Canning, 2022).

In terms of child and maternal health, the ideal maternal birth interval is neither too short or too long; an interval of two to five years is cited by some as being ideal (Ajayi & Somefun, 2020). Short birth intervals have been shown to be associated with poor child and maternal health outcomes (Conde-Agudelo et al., 2006, Conde-Agudelo et al., 2007, Conde-Agudelo et al., 2012, Tesema et al., 2023). Long birth intervals are also linked to poor child and maternal health outcomes (Cecatti, Correa-Silva, Milanez, Morais, & Souza, 2008).

While lower levels of women’s empowerment can be a cause of short birth intervals,^11^ as found in Kenya (Kudeva, Halaas, Kagotho, Lee, & Biswas, 2020), sub-optimal birth intervals can impact women’s engagement in economic activity (Finlay, 2021). Using data from 24 sub-Saharan African countries, one study showed that a greater number of young children in the household (implying short birth intervals) negatively impacts the mother’s labor force participation (de Jong, Smits, & Longwe, 2017). This finding is consistent with qualitative analysis conducted in Burundi that brought forward the idea that caring for one small child and participating in the labor force was feasible for women, but caring for two or more young children limited the mother’s time for labor force participation (Finlay and Lee, 2018, Finlay, 2021). In the study by de Jong et al. (2017), interaction effects highlight that women living in poorer districts are most impacted by the presence of small children in the household on their labor force participation. For poorer women, short birth spacing made it difficult for them to combine childcare and labor force participation. In other sub-group analysis, older women and more educated women were (slightly) more negatively affected by the presence of young children on their labor force participation than their younger and less educated counterparts. This could be due to the nature of their work – more highly educated women may be employed in jobs that have less flexibility to combine work and childcare. These empirical findings in SSA (de Jong et al., 2017) support the conjecture that healthy birth spacing is not only ideal for women’s health outcomes, but also women’s economic empowerment outcomes.

The examination of birth intervals and women’s empowerment in the sub-Saharan African context provides a different explanation of the pathway between birth intervals and empowerment than is experienced in HICs. In HICs, contraception enables women to have control over the spacing between births to minimize human capital loss (Adda et al., 2017, Gough, 2017). In the United States, Gough (2017) showed that women who have children close together and are out of the workforce for a longer period, experience the motherhood penalty – where women see their lifetime career opportunities decrease due to a longer period of interruption. In Germany (Adda et al., 2017), authors showed that longer periods out of the workplace dents a women’s career progression through skill loss, earnings loss, and a lower accumulation of experience. In summary, in HICs the narrative that explains the impact of short birth intervals on women’s labor force participation focuses on human capital loss from exiting the labor force for maternity leave, whereas, in a LMICs, where formal labor force participation is low (Bhalotra, Clarke, & Walther, 2020), the narrative centers on how longer birth intervals enable women to combine childcare and labor force participation.

The difference in results in the sub-group analysis in the de Jong et al. (2017) study points to older and more highly educated women in sub-Saharan Africa having a similar experience to women in HICs. With short birth intervals, and more than one young child to care for, older/higher educated women in sub-Saharan Africa are forced out of the labor force (they do not combine childcare and work like their younger, less-educated counterparts) and suffer the motherhood penalty to their career progression (Adda et al., 2017, Gough, 2017). The decline in their earnings then impacts gender equality in the household and dampens the woman’s economic empowerment (Williams et al., 2022).

### 3.4 The pathway linking contraceptive use to the number of children, job-type, and economic empowerment

Longitudinal data from eight Performance Monitoring and Accountability (PMA2020) sites demonstrated an increase in contraceptive prevalence among the sampled women in all sites (Ahmed et al., 2019). Cross sectional data showed a similar positive trend in the aggregate with modern contraceptive prevalence increasing from an average of about 6 percent in 1990 across 24 sub-Saharan African countries to 27 percent in 2015 (Bongaarts & Hardee, 2019). This increase in contraception within SSA was driven in part by a decline in desired family size (Motshegwa et al., 2024, Odimegwu et al., 2018). Completed fertility has also declined in most sub-Saharan African countries (Odimegwu et al., 2018), although this decline has been modest relative to countries in other continents (Bado, Guengant, & Maga, 2022).

Having fewer children has been shown to lead to greater labor force participation (Bloom et al., 2009, Clarke, 2018), but this generalized result does not easily apply in the sub-Saharan African context. According to economic theory, increasing women’s wages increases the opportunity cost of an extra child and thus reduces demand for children (lowering fertility preferences). Women who are able to realize their preferred, lower, fertility rates then use the time gained (not in childcare) to increase participation in the labor force (Galor & Weil, 1996). Empirically, however, this theory has not been realized in sub-Saharan Africa.

In a study based in Ghana, authors found that at the extensive margin (the binary outcome of whether women participate in the labor market or not) an increase in the number of children led to a decrease in labor force participation (Heath, 2017). Women who remained in the labor market after the birth of a child increased the number of hours worked (the intensive margin) (Heath, 2017). Heath (2017) suggested that this result may be because Ghanaian women considered both the time investment (raising and educating) and the monetary investment in their children. If the time investment is considered to have the highest return, then the women will not participate in the labor force and will invest her time in raising her child. If the monetary investment is considered to have the highest return, the mother will continue to participate in the labor market and use the monetary resources to invest in her child (health and schooling, for example). The choice to continue labor force participation is shown to be stronger in households with older female siblings, who are assumed able to care for the young child while the women works for monetary compensation (Heath, 2017).

In Nigeria, researchers found that the number of children has a causal impact on women’s entrepreneurship decisions (Ajefu, 2019). Defining entrepreneurship as self-employment, the authors find that a greater number of children had a significant positive impact on the likelihood of self-employment, but no significant impact in wage employment (although the coefficient is negative and significant at the 10 percent level). The increase in self-employment was particularly strong for older mothers (31–45 years old), and for those with secondary or higher education (as opposed to no education or primary) (Ajefu, 2019). The experience (that comes with age) and education enabled women to step into these self-employment roles and manage their own time juggling childcare and (self) employment (Ajefu, 2019). In terms of the education effect, another study in Nigeria found that women with less education were more likely to increase self-employment as fertility increased than women with higher levels of education (Bago & Dessy, 2023). Interestingly, in the examination of time use data it was found that “although extensive margin participation of women is high in SSA, women tend to work in the market for only a few hours each week, with the rest of their work hours spent in home production” (Dinkelman & Ngai, 2022).

Employment and empowerment are related (McKelway, 2022) for women in sub-Saharan Africa, but having a job or a source of income is not synonymous to economic empowerment (Finlay et al., 2018, Verick, 2014). Job-type is an important factor in determining if employment is empowering for women (Verick, 2014). For women in sub-Saharan Africa, the switch to self-employment at the event of a birth can be an indication of a decrease in empowerment. The switch to self-employment comes with potentially lower wages (resources), a more limited choice-set (lower agency) and a revision of her self-determined achievements. They type of employment a woman has is an important indicator of her empowerment trajectory (Finlay et al., 2018, Verick, 2014).

To summarize, the pathway linking contraceptive use to the number of children and economic empowerment, in SSA, higher numbers of children can lead to greater labor force participation, but the type of employment is not necessarily contributing positively to the women’s economic empowerment process. The increase in labor force participation has been explained as women valuing the monetary investment in their children over the investment of her time raising the child (Heath, 2017), or women increasing their participation in self-employment activities (Ajefu, 2019, Bago and Dessy, 2023) that can imply working fewer hours (Dinkelman & Ngai, 2022) or combining childcare and economic activity. The switch to self-employment may bring greater flexibility to combine childcare and economic activity, but it does not imply greater economic empowerment.

## 4 Discussion

In this paper, we provided a narrative review of the literature that explored the long-reaching connection from contraception to women’s economic empowerment. Investing in contraception is an effective way of elevating women’s economic empowerment, particularly when those investments are made in adolescent girls so that the benefits of the investment in contraception are realized over a long period of time (Miller, 2010). In LMICs, access to and use of contraception by women has been shown to have a direct, and an indirect, effect on women’s economic empowerment. The direct effect comes from greater bodily autonomy and contraception enabling greater decision-making power (Alano and Hanson, 2018, Dhak et al., 2020, Lee-Rife, 2010). The indirect effect comes from access to and use of contraception enabling control over fertility; that is timing (Ardington et al., 2015), spacing (de Jong et al., 2017) and limiting (Heath, 2017). With access to contraception, women can avoid unwanted teen pregnancy and stay in school (Ardington et al., 2015) leading to greater school completion rates and higher paying jobs (Herrera et al., 2019). Short birth intervals, implying more than one young child to care for, impacts job type and work intensity for women in LMICs (de Jong et al., 2017). In LMICs, total number of children can impact job type (such as self-employment), increase a woman’s labor force participation, or change the number of hours she works – with mother’s calculating a complex trade-off of monetary and time investments in children (Heath, 2017). In the sub-Saharan African context, work and income do not equate to women’s economic empowerment, and having decision-making power over earnings is a key element in interpreting employment as economic empowerment (Soharwardi & Ahmad, 2020).

## 5 Social norms of gender discrimination

The impact of contraception on women’s economic empowerment is hampered by social norms of gender-based discrimination and inequality to varying degrees across contexts. Women’s empowerment cannot happen without the accompanying social change (Kabeer, 1999). The transformed woman must be valued and respected for her empowered state (Baird et al., 2015, Baird et al., 2016, Goldin and Katz, 2002). If not, the perceived power imbalance caused by her rise towards equality may threaten the relative power of the superior (her husband, father-in-law, or head of household) (Akerlof & Kranton, 2000). While there is a pathway for women who are economically empowered to contribute to economic development, gender based discrimination can hold them back and limit the possibility of their economic success (Doepke and Tertilt, 2019, Duflo, 2012). For example, if women are limited in their education attainment, they will not have this initial comparative advantage for service-based jobs and they are disadvantaged from the outset (Santos Silva & Klasen, 2021). In a paper that linked women’s economic empowerment to economic development, the conclusion was that in the microeconomic evidence to date, investment in women’s economic empowerment did not lead to as great an advancement in economic development as investment in men’s economic empowerment because gender based discrimination limited the returns on investment for women compared to men (Doepke and Tertilt, 2019, Duflo, 2012). Structural barriers mean that women have faced greater hurdles in gaining permits, accessing trading markets, interacting with buyers, and these gender biases accumulated to dampen the return on investment for women compared to the return on investment for men (Duflo, 2012).

Furthermore, during the empowerment process women may be subject to the threat of violence (intimate partner violence, domestic violence, gender-based violence), which can alter a women’s preferences and ability to achieve her own goals. The threat of violence is pervasive and is a strong current in affecting the process of empowerment for women (Abramsky et al., 2019, Chakraborty et al., 2018, Heath, 2014, MacGregor et al., 2021, Stöckl et al., 2021, Vyas and Watts, 2009, Vyas et al., 2015). Policies that help women circumvent barriers that prevent her access to labor force participation, and policies that dismantle discrimination, are both useful in enabling women to participate in economic activity (Jayachandran, 2020). Thus, streamlining the positive impact women’s economic participation can have on economic growth. Policies that help women overcome discrimination (Jayachandran, 2020) that limits their contribution to the economy will not only pave the way for their own economic empowerment, but also enable women to contribute to the national economy. Programs that dismantle discriminatory gender norms (Dhar, Jain, & Jayachandran, 2022), are also important in facilitating women’s economic participation – enabling her spend her time in education and working if she desires.

Context matters for implementation of programs to increase contraceptive use (Ali et al., 2023, Weinberger et al., 2019). In contexts where deeply ingrained social and cultural norms perpetuate gender inequality, strategies may focus on community mobilization, awareness-raising, and behavior change campaigns. Engaging with religious and community leaders, working through community-based organizations, and promoting positive role models can be effective approaches. This then paves the way for improving gender equality in access to education and training and labor force participation and entrepreneurship. Similar to Duflo, 2012, Kabeer, 2005 also called out the need for there to be an underlying drive for gender equality and reducing gender bias, so that women and girls can have access to quality education, work and contribute as equals to the economy as a whole.

### 5.1 Reverse causality, empowerment to contraceptive use

In the case of adolescents, a systematic review found that the literature focused on the impact of empowerment on contraceptive use (Lassi et al., 2024). Of the 40 studies found in this systematic review, 26 were randomized control trials that showed a significant effect of sexual and reproductive empowerment in increasing ever use of contraception (Lassi et al., 2024). In another systematic review, it was found that reproductive empowerment leads to increased contraceptive use among all women of reproductive age (Burke et al., 2021). Economic empowerment has also been shown to have a positive impact on increasing contraceptive use among women of reproductive age (Reed, West, Salazar, & Monroy, 2018). In studies that examine the effect of contraceptive use on women’s economic empowerment, controlling for this evident reverse causality is critical.

To control for reverse causality, the use of randomized control trials has become a gold standard (Finlay & Lee, 2018), but in the case of examining the effects of contraceptive use on women’s economic empowerment the time lapse is too long, and effects of the interventions will be diluted over these years. Taking a modeling approach to the question of the investment case for contraceptive use in promoting women’s economic empowerment, is a more time- and resource-efficient approach. A Bayesian non-parametric model (Garraza, Lucas, & Speizer, and Leontine Alkema, 2024), causal analysis across the life course (Seager, Hamory, Parisotto, & Baird, 2024), fictive cohorts and sequence analysis (Zan, Rossier, Studer, Dao, & Guiella, 2024), and agent-based models (Zimmermann, Sanz-Leon, Stuart, Driano, & O’Brien, 2024), are examples of how this modeling approach can be applied.

Other factors, in addition to contraception, play a role in the empowerment process in sub-Saharan Africa. Prevention of intimate partner violence is a key factor in supporting women’s economic empowerment (Stöckl et al., 2021), and education programs have been instrumental (Duflo et al., 2015). Cash transfers to enable education (Baird et al., 2015), teen well-being (Özler et al., 2020), or delayed marriage (Yount, Crandall, & Cheong, 2018) have also been implemented. The purpose of this paper was not to show the relative magnitude of the role of contraception in boosting women’s economic empowerment compared to these other interventions, but to reinforce the evidence that contraception is a pathway to women’s economic empowerment along with these other interventions, and the importance of the pathway of reproductive empowerment to economic empowerment for women.

### 5.2 Limitations

This paper was narrow in its scope, focusing on showcasing the existing peer-review literature that addressed the topic of the causal impact of contraceptive access and use on women’s economic empowerment in LMICs, particularly in SSA. Literature searches were conducted in English and limited to articles published in English. This meant that French language studies that consider the SSA case were not included. Furthermore, as a narrative review and not a systematic review, some articles may have been unintentionally omitted.

## 6 Policy implications

Applying policies and programs that were successful in one locale to another would be unwise without consideration of the new context. As such, using results in this review to develop policy or implement programs must be done with care. A robust understanding of the social norms and attitudes towards reproductive empowerment and women’s economic empowerment in the target context is an essential starting point. Much of the existing empowerment literature in LMICs has focused on South Asia. Within this context, one of the key indicators of low empowerment is lack of mobility – women cannot leave the house without their husbands or a chaperone, making work, purchasing decisions, and seeking healthcare close to impossible. The empowerment process in South Asia thus focuses on overcoming this limitation on women’s mobility. Comparatively in SSA, there is a growing body of research in SSA that demonstrates great diversity in empowerment across the continent (O’Brien et al., 2023), but one key indicator of low empowerment may be the poverty trap (Wanjala, 2021). Key limitations, social norms, available infrastructure, all differ across contexts, and shape what policies and programs would effectively enable contraceptive access and use and lead to positive improvement in women’s economic empowerment.

To date, public messaging around contraceptive access and use in sub-Saharan Africa has focused on its merits in controlling birth spacing. The limited (but positive) research on this pathway to empowerment (de Jong et al., 2017), and number of children to empowerment, both (Heath, 2017) stop at the examination of impacts on women’s labor force participation. While these studies offer valuable insight into positive impacts on women’s relative earnings (Williams et al., 2022) and job-type (McKelway, 2022), these “work” outcomes do not fully capture women’s economic empowerment (Finlay et al., 2018, McKelway, 2022, Verick, 2014). For example, the rise in self-employment that is observed in mothers in Nigeria (Ajefu, 2019) does not necessarily positively correlate to the elements of empowerment. Self-employment may bring greater control over hours worked and workplace conditions (for example, combining childcare and work), but there may be a reduction in income (intentionally or unintentionally), a reduction in the choice-set available to women (a reduction in agency), and her self-defined achievements may be less ambitious. In the case of the birth-spacing and number of children pathways, contraceptive access and use enables women to better plan their work-lives, but the improvements offered by this planning is incremental improvements.

Most notably, across contexts, the literature in this review suggests that investment in contraception access and use is particularly transformative for adolescent girls and young women (Miller, 2010). Using contraception to delay childbearing enables them to use their time for education (Ardington et al., 2015), skill building, and job opportunities in the formal sector (Herrera et al., 2019), and positions them with greater gender equality in their future household and greater decision-making power (Anderson et al., 2017). Thus, elucidating a strong investment case for contraceptive access and use as a pathway to supporting empowerment in adolescents and young women.

## 7 Conclusion

Enabling reproductive empowerment – through contraceptive access and use – can contribute to advancing women’s economic empowerment. The seminal thesis that contraception enables women to plan their reproductive lives, and subsequently their economic lives, holds (Goldin & Katz, 2002). Evidence from many contexts – different countries, different ages of women, different education levels – corroborates positive indirect and direct pathways from contraception to economic empowerment. Particularly transformative, and long-lasting, effects have been shown in adolescents and young women. To maximize potential impact, care must be taken to adapt promising policies and programs across contexts, and address potential modifying factors (e.g., discrimination and violence). This evidence in this review supports the notion that investing in programs that promote – and advancing policies that support – contraceptive use and access across the reproductive life course can yield benefits to economic empowerment.

## CRediT authorship contribution statement

**Jocelyn E. Finlay:** Writing – review & editing, Validation, Resources, Methodology, Funding acquisition, Data curation, Writing – original draft, Supervision, Project administration, Investigation, Formal analysis, Conceptualization. **Mariam Gulaid:** Writing – original draft, Validation, Software, Project administration, Investigation, Formal analysis, Writing – review & editing, Visualization, Supervision, Resources, Methodology, Funding acquisition, Conceptualization. **Chiseche Mibenge:** Writing – review & editing, Visualization, Supervision, Resources, Methodology, Funding acquisition, Conceptualization, Writing – original draft, Validation, Software, Project administration, Investigation, Formal analysis. **Nyovani Madise:** Writing – review & editing, Methodology, Formal analysis, Writing – original draft, Investigation, Conceptualization. **Naa Dodua Dodoo:** Writing – review & editing, Methodology, Formal analysis, Writing – original draft, Investigation, Conceptualization. **John Stover:** Writing – original draft, Formal analysis, Writing – review & editing, Investigation, Conceptualization. **Michelle Weinberger:** Writing – review & editing, Investigation, Conceptualization, Writing – original draft, Formal analysis. **Michelle O’Brien:** Writing – review & editing, Validation, Formal analysis, Writing – original draft, Investigation, Conceptualization. **Marita Zimmermann:** Writing – review & editing, Validation, Formal analysis, Writing – original draft, Investigation, Conceptualization.

## Declaration of competing interest

The authors declare the following financial interests/personal relationships which may be considered as potential competing interests: Jocelyn E. Finlay PhD: Declares no competing interests.

Mariam Gulaid, MPH: Declares no competing interests.

Chiseche Mibenge PhD: Declares no competing interests.

Nyovani Madise PhD: Declares no competing interests.

Naa Dodua Dodoo PhD: Declares no competing interests.

John Stover, MA: Declares no competing interests.

Michelle Weinberger, MSc: Declares no competing interests.

Michelle O’Brien PhD: Declares that the donor (BMGF) is also their employer.

Marita Zimmermann MPH PhD: Declares that the donor (BMGF) is also their employer.

## Data Availability

No numeric data was used for the research described in the article.

## Footnotes

1 Also see the Gender Inequality Index that shows the range if Gender Inequality in the most recent year is the same across South Asia and sub-Saharan Africa Gender Inequality Index | Human Development Reports (undp.org).

2 In this paper we focus on contraceptive access and use, an element of reproductive empowerment. In this section, we discuss how the broader concept of reproductive empowerment connects to economic empowerment, but the paper focuses in on a subsection of reproductive empowerment with contraceptive access and use. We do not assume that reproductive empowerment and contraceptive access and use are synonymous, but rather contraceptive access and use is a part of reproductive empowerment.

3 A woman is economically empowered when she has both the ability to succeed and advance economically and the power to make and act on economic decisions (Anne Marie Golla, Anju Malhotra, Priya Nanda, and Rekha Mehra, 2011).

4 Cai and Pleskac (2020) includes structural institutions, laws, as resources necessary for empowerment.

5 It is important to note that the transformative nature of the empowerment process differentiates it from ‘efficacy’ (Kabeer, 1999). If a woman gains resources and agency to execute on her gendered roles and responsibilities more effectively (looking after her children, cooking more nutritious meals for the family, for example), this gain is not ‘empowering’ for women. For a woman’s actions to be within the empowerment process, they must be transformative – her lifelong welfare must be improved through an elevation of self-determination.

6 Equality and women’s empowerment can be perceived to erode the social order and trust in relationships.

7 The WEAI is a survey tool composed of two sub-indices: one measures women’s empowerment across five domains in agriculture, and the other measures gender parity in empowerment within the household. The tool also measures women’s empowerment relative to men within their households. Data has been collected in sites to validate this tool (Quisumbing et al., 2023).

8 We reference four papers published before 2002 (Akerlof & Kranton, 2000; Barham et al., 1998; Galor & Weil, 1996; Kabeer, 1999) because they illustrate the theoretical foundation of the connection between contraception and women’s economic empowerment (rather than for their empirical results).

9 46 percent of resident women in the study area age 20–50 had had their first birth before the age of 20.

10 It is important to note that in South Africa, pregnant girls cannot be expelled from school (Atake & Ali, 2015). On the other hand, in other sub-Saharan African countries, such as Sierre Leone, Tanzania and Equatorial Guinea, pregnant girls can be expelled and the consequence of both lower education and caring for a child are borne across the life course (Gunther & Harttgen, 2022) in these countries.

11 This is the reverse causal pathway of fertility to empowerment, which is not central to this narrative review.

## References

[b0005] Abramsky T., Lees S., Stöckl H., Harvey S., Kapinga I., Ranganathan M., Mshana G., Kapiga S. (2019). Women’s income and risk of intimate partner violence: Secondary findings from the MAISHA cluster randomised trial in North-Western Tanzania. BMC Public Health.

[b0010] Adda J., Dustmann C., Stevens K. (2017). The career costs of children. Journal of Political Economy.

[b0015] Ahmed S., Choi Y., Rimon J.G., Alzouma S., Gichangi P., Guiella G., Kayembe P., Kibira S.P., Makumbi F., OlaOlorun F., Omoluabi E., Otupiri E., Oumarou S., Seme A., Shiferaw S., Anglewicz P., Radloff S., Tsui A. (2019). Trends in contraceptive prevalence rates in Sub-Saharan Africa since the 2012 London summit on family planning: results from repeated cross-sectional surveys. The Lancet Global Health.

[b0020] Ajayi A.I., Somefun O.D. (2020). Patterns and determinants of short and long birth intervals among women in selected Sub-Saharan African Countries. Medicine.

[b0025] Ajefu J.B. (2019). Does having children affect women’s entrepreneurship decision? Evidence from Nigeria. Review of Economics of the Household.

[b0030] Akerlof G.A., Kranton R.E. (2000). Economics and Identity. The Quarterly Journal of Economics.

[b0035] Alano A., Hanson L. (2018). Women’s perception about contraceptive use benefits towards empowerment: A phenomenological study in Southern Ethiopia. PLoS One1.

[b0040] Ali M., Kiarie J., Shah I. (2023). Priorities for research on family planning impact: recommendations of a WHO think tank meeting. Family Medicine and Community Health.

[b0045] Alkire S., Meinzen-Dick R., Peterman A., Quisumbing A., Seymour G., Vaz A. (2013). The women’s empowerment in agriculture index. World Development.

[b0050] Anderson C.L., Reynolds T.W., Gugerty M.K. (2017). Husband and wife perspectives on farm household decision-making authority and evidence on intra-household accord in rural Tanzania. World Development.

[b0055] Annan J., Donald A., Goldstein M., Martinez P.G., Koolwal G. (2021). Taking power: Women’s empowerment and household well-being in sub-Saharan Africa. World Development.

[b0060] Ardington C., Menendez A., Mutevedzi T. (2015). Early childbearing, human capital attainment, and mortality risk: evidence from a longitudinal demographic surveillance area in Rural KwaZulu-Natal, South Africa. Economic Development and Cultural Change.

[b0065] Atake E.-H., Ali P.G. (2019). Women’s empowerment and fertility preferences in high fertility countries in Sub-Saharan Africa. BMC Women’s Health.

[b0070] Bado A.R., Guengant J.-P., Maga H.I., May J.F., Goldstone J.A. (2022). International handbook of population policies.

[b0075] Bago J.L., Dessy S.E. (2023). Motherhood and women’s self-employment: Theory and evidence from Nigeria. Economic Development and Cultural Change.

[b0080] Bailey M. (2006). More power to the pill: The impact of contraceptive freedom on women’s life cycle labor supply. The Quarterly Journal of Economics.

[b0085] Bailey M. (2013). Fifty years of family planning: New evidence on the long-run effects of increasing access to contraception. Brookings Papers on Economic Activity.

[b0090] Bailey M., Hershbein B., Miller A. (2012). The opt-in revolution? Contraception and the gender gap in wages. American Economic Journal: Applied Economics.

[b0095] Baird S., Chirwa E., McIntosh C., Özler B. (2015). 3ie Impact Evaluation Report 27.

[b0100] Baird, S., McIntosh, C., & Ozler, B. (2016). “When the Money Runs Out: Do Cash Transfers Have Sustained Effects on Human Capital Accumulation?” *World Bank Policy Research Working Paper* 7901.

[b0105] Balasubramanian P., Ibanez M., Khan S., Sahoo S. (2024). Does women’s economic empowerment promote human development in low- and middle-income countries? A meta-analysis. World Development.

[b0110] Bandiera O., Buehren N., Burgess R., Goldstein M., Gulesci S., Rasul I., Sulaiman M. (2020). Women’s empowerment in action: Evidence from a randomized control trial in Africa. American Economic Journal: Applied Economics.

[b0115] Banjo O.O. (2024). Women’s empowerment and completed fertility in Nigeria: What is the modulating effect of religion on the relationship?. Women’s Reproductive Health.

[b0120] Bankole A., Singh S. (1998). Couples’ fertility and contraceptive decision-making in developing countries: Hearing the man’s voice. International Family Planning Perspectives.

[b0125] Barham T., Champion B., Foster A.D., Hamadani J.D., Jochem W.C., Kagy G., Kuhn R., Menken J., Razzaque A., Root E.D., Turner P.S. (2021). Thirty-five years later: Long-term effects of the matlab maternal and child health/family planning program on older women’s well-being. Proceedings of the National Academy of Sciences.

[b0130] Becker G.S., Gregg Lewis H. (1973). On the interaction between the quantity and quality of children. Journal of Political Economy.

[b0135] Bedigen W. (2022). Indigenous South Sudanese understanding of women empowerment. World Development Perspectives.

[b0140] Bhalotra S., Clarke D., Walther S. (2020).

[b0145] Black S.E., Devereux P.J., Salvanes K.G. (2008). Staying in the classroom and out of the maternity ward? The effect of compulsory schooling laws on teenage births. The Economic Journal.

[b0150] Bloom D.E., Canning D., Fink G., Finlay J.E. (2009). Fertility, female labor force participation, and the demographic dividend. Journal of Economic Growth.

[b0155] Bongaarts J. (2024). Fertility transitions in low- and middle-income countries: The role of preferences. Population and Development Review.

[b0160] Bongaarts J., Hardee K. (2019). Trends in contraceptive prevalence in Sub-Saharan Africa: The roles of family planning programs and education. African Journal of Reproductive Health.

[b0165] Brown K., Levison D., Maynes M.J., Vavrus F. (2021). Children and youth as subjects, objects, agents: Innovative approaches to research across space and time.

[b0170] Burke H.M., Ridgeway K., Murray K., Mickler A., Thomas R., Williams K. (2021). Reproductive empowerment and contraceptive self-care: A systematic review. Sexual and Reproductive Health Matters.

[b0175] Buvinic M., Gupta M.D., Casabonne U. (2009). Gender, poverty and demography: An overview. The World Bank Economic Review.

[b0180] Buvinic M., O’Donnell M., Knowles J.C., Bourgault S. (2020).

[b0185] Cai X., Pleskac T.J. (2023). When alternative hypotheses shape your beliefs: Context effects in probability judgments. Cognition.

[b0190] Canning D., Schultz T.P. (2012). The economic consequences of reproductive health and family planning. The Lancet.

[b0195] Cecatti J.G., Correa-Silva E.P.B., Milanez H., Morais S.S., Souza J.P. (2008). The associations between inter-pregnancy interval and maternal and neonatal outcomes in Brazil. Maternal and Child Health Journal.

[b0200] Chakraborty T., Mukherjee A., Rachapalli S.R., Saha S. (2018). Stigma of sexual violence and women’s decision to work. World Development.

[b0205] Church A.C., Ibitoye M., Chettri S., Casterline J.B. (2023). Traditional supports and contemporary disrupters of high fertility desires in Sub-Saharan Africa: A scoping review. Reproductive Health.

[b0210] Clarke D. (2018). Children and their parents: A review of fertility and causality. Journal of Economic Surveys.

[b0215] Conde-Agudelo A., Rosas-Bermudez A., Castaño F., Norton M.H. (2012). Effects of birth spacing on maternal, perinatal, infant, and child health: A systematic review of causal mechanisms. Studies in Family Planning.

[b0220] Conde-Agudelo A., Rosas-Bermúdez A., Kafury-Goeta A.C. (2006). Birth spacing and risk of adverse perinatal outcomes a meta-analysis. Journal of the American Medical Association.

[b0225] Conde-Agudelo A., Rosas-Bermúdez A., Kafury-Goeta A.C. (2007). Effects of birth spacing on maternal health: A systematic review. American Journal of Obstetrics & Gynecology.

[b0230] Condren M., Allison S.T., Beggan J.K., Goethals G.R. (2024). Encyclopedia of Heroism Studies.

[b0235] Dadzie L.K., Yengnone H., Frimpong J.B., Agbaglo E., Seidu A.-A., Ahinkorah B.O. (2024). Association between women’s empowerment and fertility preferences in Ghana. International Health.

[b0240] de Jong E., Smits J., Longwe A. (2017). Estimating the causal effect of fertility on women’s employment in Africa using twins. World Development.

[b0245] Debpuur C., Phillips J.F., Jackson E.F., Nazzar A., Binka F.N. (2002). The impact of the navrongo project on contraceptive knowledge and use, reproductive preferences, and fertility. Studies in Family Planning.

[b0250] Despard M.R., Friedline T., Martin-West S. (2020). Why do households lack emergency savings? The role of financial capability. Journal of Family and Economic Issues.

[b0255] Dhak B., Saggurti N., Ram F. (2020). Contraceptive use and its effect on Indian women’s empowerment: Evidence from the national family health survey-4. Journal of Biosocial Science.

[b0260] Dhar D., Jain T., Jayachandran S. (2022). Reshaping adolescents’ gender attitudes: Evidence from a school-based experiment in India. American Economic Review.

[b0265] Dinkelman T., Ngai L.R. (2022). Time use and gender in Africa in times of structural transformation. Journal of Economic Perspectives.

[b0270] Doepke M., Tertilt M. (2019). Does female empowerment promote economic development?. Journal of Economic Growth.

[b0275] Duflo E. (2012). Women empowerment and economic development. Journal of Economic Literature.

[b0280] Duflo E., Dupas P., Kremer M. (2015). Education, HIV, and early fertility: experimental evidence from Kenya. American Economic Review.

[b0285] Duflo E., Udry C. (2004). National Bureau of Economic Research Working Paper Series 10498.

[bib631] Dupas P., Jayachandran S., Lleras-Muney A., Rossi P. (2025). The Negligible Effect of Free Contraception on Fertility: Experimental Evidence from Burkina Faso. American Economic Review.

[b0290] Earle A., Raub A., Sprague A., Heymann J. (2023). Progress towards gender equality in paid parental leave: An analysis of legislation in 193 countries from 1995–2022. Community, Work & Family.

[b0295] Esquivel V., Sweetman C. (2016). INTRODUCTION: Gender and the sustainable development goals. Gender and Development.

[b0300] Ewerling F., Raj A., Victora C.G., Hellwig F., Coll C.V., Barros A.J. (2020). SWPER global: A survey-based women’s empowerment index expanded from africa to all low- and middle-income countries. Journal of Global Health.

[b0305] Finlay J.E. (2021). Women’s reproductive health and economic activity: A narrative review. World Development.

[b0310] Finlay J.E., Efevbera Y., Ndikubagenzi J., Karra M., Canning D. (2018). Reframing the measurement of women’s work in the Sub-Saharan African context. Work, Employment and Society First Online.

[b0315] Finlay J.E., Lee M.A. (2018). Identifying causal effects of reproductive health improvements on women’s economic empowerment through the population poverty research initiative. The Milbank Quarterly.

[b0320] Galor O., Weil D.N. (1996). The gender gap, fertility, and growth. The American Economic Review.

[b0325] Gammage S., Joshi S., van der Meulen Rodgers Y. (2020). The Intersections of Women’s Economic and Reproductive Empowerment. Feminist Economics.

[b0330] Godoy Garraza L., Speizer I.S., Alkema L. (2024). How to Estimate causal effects associated with family planning? An introduction to prince BART, a new approach to effect estimation based on principal stratification and Bayesian non-parametric models [Version 2]. VeriXiv.

[b0335] Global Forum on Innovation in Health Professional Education. (2015). *Empowering Women and Strengthening Health Systems and Services Through Investing in Nursing and Midwifery Enterprise: Lessons from Lower-Income Countries: Workshop Summary*. Washington (DC). 26225403

[b0340] Goldin C., Katz L.F. (2002). The power of the pill: Oral contraceptives and women’s career and marriage decisions. Journal of Political Economy.

[b0345] Golla A.M., Malhotra A., Nanda P., Mehra R. (2011). Understanding and measuring women’s economic empowerment. ICRW.

[b0350] Gough M. (2017). Birth spacing, human capital, and the motherhood penalty at midlife in the United States. Demographic Research.

[b0355] Gudu W., Bekele D., Surur F., Teklu A., Abesha Y., Nigatu B., Kassa M., Lako T.K., Sium A.F. (2023). Role of male partner in women’s fertility decision and family planning utilization in four regional states of Ethiopia: A mixed-method study. International Journal of Gynecology & Obstetrics.

[b0360] Gunther I., Harttgen K. (2016). Desired fertility and number of children born across time and space. Demography.

[b0370] Heath R. (2014). Women’s access to labor market opportunities, control of household resources, and domestic violence: Evidence from Bangladesh. World Development.

[b0375] Heath R. (2017). Fertility at work: Children and women’s labor market outcomes in Urban Ghana. Journal of Development Economics.

[b0380] Herrera Almanza C., Sahn D.E. (2018). Early childbearing, school attainment, and cognitive skills: Evidence from madagascar. Demography.

[b0385] Herrera C., Sahn D.E., Villa K.M. (2019). Teen Fertility and female employment outcomes: Evidence from Madagascar. Journal of African Economies.

[b0390] Jayachandran, S. (2020). “Social Norms as a Barrier to Women’s Employment in Developing Countries.” *National Bureau of Economic Research Working Paper Series* 27449. doi: 10.3386/w27449.

[b0395] Joshi, S., & Schultz, T. P. (2007). “Family planning as an investment in development: evaluation of a program’s consequences in Matlab, Bangladesh.” *Economic Growth Center Yale University Center Discussion Paper* 951.

[b0400] Joshi S., Paul Schultz T. (2013). Family planning and women’s and children’s health: Long-term consequences of an outreach program in matlab, Bangladesh. Demography.

[b0405] Kabeer N. (1999). Resources, agency, achievements: reflections on the measurement of women’s empowerment. Development and Change.

[b0410] Kabeer N. (2005). Gender equality and women’s empowerment: A critical analysis of the third millennium development goal 1. Gender & Development.

[b0415] Kabeer N. (2015). Gender, poverty, and Inequality: A brief history of feminist contributions in the field of international development. Gender & Development.

[b0420] Karra M., David Canning M.Q., Guo B.N., Canning D. (2022). The causal effect of a family planning intervention on women’s contraceptive use and birth spacing. Proceedings of the National Academy of Sciences of the United States of America.

[b0425] Kudeva R., Halaas B., Kagotho N., Lee G., Biswas B. (2020). Optimal birth interval and empowerment: A closer look at women’s agency in Kenya. African Journal of Midwifery and Women’s Health.

[b0430] Lassi Z.S., Rahim K.A., Stavropoulos A.M., Ryan L.M., Tyagi J., Adewale B., Kurji J., Bhaumik S., Meherali S., Ali M. (2024). Use of contraceptives, empowerment and agency of adolescent girls and young women: A systematic review and meta-analysis. BMJ Sexual and Reproductive Health.

[b0435] Laszlo S., Grantham K., Oskay E., Zhang T. (2020). Grappling with the challenges of measuring women’s economic empowerment in intrahousehold settings. World Development.

[b0440] Lee-Rife S.M. (2010). Women’s empowerment and reproductive experiences over the lifecourse. Social Science & Medicine.

[b0445] Liu R.W., Lapinski M.K. (2024). Cultural influences on the effects of social norm appeals. Philosophical Transactions of the Royal Society B: Biological Sciences.

[b0450] MacGregor J.C.D., Oliver C.L., MacQuarrie B.J., Nadine Wathen C. (2021). Intimate partner violence and work: A scoping review of published research. Trauma, Violence, & Abuse.

[b0455] Mare K.U., Sabo K.G., Mohammed A.A., Leyto S.M., Mulaw G.F., Tebeje T.M., Aychiluhm S.B., Ebrahim O.A., Wuneh A.G., Seifu B.L. (2023). Suboptimal birth spacing practice and its predictors among reproductive-age women in Sub-Saharan African countries: A multilevel mixed-effects modeling with robust Poisson regression. Reproductive Health.

[b0460] May J.F. (2017). The politics of family planning policies and programs in Sub-Saharan Africa. Population and Development Review.

[b0465] McKelway M. (2022). Women’s employment and empowerment: descriptive evidence. AEA Papers and Proceedings.

[b0470] Miedema S.S., Haardörfer R., Girard A.W., Yount K.M. (2018). Women’s empowerment in East Africa: Development of a cross-country comparable measure. World Development.

[b0475] Miller G. (2010). Contraception as development? New evidence from family planning in Colombia. The Economic Journal.

[b0480] Motshegwa G., Tsawe M., Legotlo O., Malatji M., Mathabatha S., Phateng R., Tshabalala K.P., Seima J., Berries M., Seokamo T. (2024). Factors determining intention to use contraception among sexually active women in South Africa: A multilevel modelling approach. Advances in Public Health.

[b0485] Musonera A., Heshmati A., Heshmati A. (2017). Studies on economic development and growth in selected African countries.

[b0490] Nabimanya, B., Mayanja, E., Kyarikunda, M., et al. (2024). Empowering women economically is more important than personal and socio-cultural empowerment. Analysis of 2022 Kenya Demographic and Health Survey, PREPRINT (Version 1). 10.1186/s12905-025-04082-7 PMC12584310 41188764

[b0495] Nguyen-Phung H.T., Nthenya N.N. (2024). The causal effect of education on women’s empowerment: Evidence from Kenya. Education Economics.

[b0500] O’Brien M.L., Zimmermann M., Eitmann L., Chao D.L., Proctor J.L. (2023). Contraceptive adoption and changes in empowerment in Kenya, Nigeria, and Senegal. Studies in Family Planning.

[b0505] Odimegwu C.O., Akinyemi J.O., Banjo O.O., Olamijuwon E., Amoo E.O. (2018). Fertility, family size preference and contraceptive use in sub-Saharan Africa: 1990-2014. African Journal of Reproductive Health.

[b0510] Olu-Owolabi F.E., Amoo E., Samuel O., Oyeyemi A., Adejumo G. (2020). Female-dominated informal labour sector and family (in) stability: The interface between reproduction and production edited by L. Geraghty. Cogent Arts & Humanities.

[b0515] Onyina K., Baye R.S. (2024). Impact of social protection policies on inclusive growth in sub-Saharan Africa: evidence from bias-corrected dynamic panel. Cogent Economics & Finance.

[b0520] Özler B., Hallman K., Guimond M.-F., Kelvin E.A., Rogers M., Karnley E. (2020). Girl empower – A gender transformative mentoring and cash transfer intervention to promote adolescent wellbeing: impact findings from a cluster-randomized controlled trial in Liberia. SSM – Population Health.

[b0525] Pande R.P., Astone N.M. (2007). Explaining son preference in rural India: The independent role of structural versus individual factors. Population Research and Policy Review.

[b0530] Pradhan M.R., Unisa S., Rawat R., Surabhi S., Saraswat A., Reshmi R.S., Sethi V. (2023). Women empowerment through involvement in community-based health and nutrition interventions: evidence from a qualitative study in India. PLoS One.

[b0535] Quisumbing A., Cole S., Elias M., Faas S., Galiè A., Malapit H., Meinzen-Dick R., Myers E., Seymour G., Twyman J. (2023). Measuring women’s empowerment in agriculture: Innovations and evidence. Global Food Security.

[b0540] Raj A., Dey A., Rao N., Yore J., McDougal L., Bhan N., Silverman J.G., Hay K., Thomas E.E., Fotso J.C., Lundgren R. (2024). The EMERGE framework to measure empowerment for health and development. Social Science & Medicine (1982).

[b0545] Raj, A., Dey, A. K., Lundgren, R., and the EMERGE team. (2021). *A Conceptual Framework for Measuring Women’s Empowerment. Evidence-Based Measures of Empowerment for Research on Gender Equality [EMERGE]*. San Diego, CA: Center on Gender Health and Equity (GEH) University of California San Diego.

[b0550] Reed, E., West, B. S., Salazar, M., & Monroy, R. V. (2018). Economic empowerment to improve sexual and reproductive health among women and girls. *Global Perspectives on Women’s Sexual and Reproductive Health Across the Lifecourse*, edited by S. Choudhury, J. T. Erausquin, and M. Withers. Cham: Springer International Publishing. pp. 297–315.

[b0555] Ruthbah U. (2020). Does lower fertility empower women? Evidence from rural Bangladesh. Economics & Human Biology.

[b0560] Santos Silva M., Klasen S. (2021). Gender inequality as a barrier to economic growth: A review of the theoretical literature. Review of Economics of the Household.

[b0565] Seager J., Hamory J., Parisotto L., Baird S. (2024). Does family planning improve female economic empowerment in Sub-Saharan Africa: Conceptual framework and protocol for causal analysis across the life course [Version 1]. VeriXiv.

[b0570] Soharwardi M.A., Ahmad T.I. (2020). Dimensions and determinants of women empowerment in developing countries. International Journal of Sustainable Development and Planning.

[b0575] Stöckl H., Hassan A., Ranganathan M., Hatcher A.M. (2021). Economic empowerment and intimate partner violence: A secondary data analysis of the cross-sectional demographic health surveys in Sub-Saharan Africa. BMC Women’s Health.

[b0580] Tesema G.A., Teshale A.B., Yeshaw Y., Angaw D.A., Molla A.L. (2023). Assessing the effects of duration of birth interval on adverse pregnancy outcomes in Sub-Saharan Africa: A propensity score-matched analysis. BMJ Open.

[b0585] Verick S. (2014). Female labor force participation in developing countries. IZA World of Labor.

[b0590] Vyas S., Mbwambo J., Heise L. (2015). Women’s paid work and intimate partner violence: Insights from Tanzania. Feminist Economics.

[b0595] Vyas S., Watts C. (2009). How does economic empowerment affect women’s risk of Intimate partner violence in low and middle income countries? A systematic review of published evidence. Journal of International Development.

[b0600] Wanjala, B. M. (2021). Women, poverty, and empowerment in Africa. pp. 1657–79 in *The Palgrave Handbook of African Women’s Studies*, edited by O. Yacob-Haliso and T. Falola. Cham: Springer International Publishing.

[b0605] Weinberger M., Williamson J., Stover J., Sonneveldt E. (2019). Using evidence to drive impact: Developing the FP goals impact matrix. Studies in Family Planning.

[b0610] Williams M.J. (2020). External validity and policy adaptation: From impact evaluation to policy design. The World Bank Research Observer.

[b0615] Williams E.M., Väisänen H., Padmadas S.S. (2022). Women’s economic empowerment in Sub-Saharan Africa. Demographic Research.

[b0620] Yount K.M., Crandall A.A., Cheong Y.F. (2018). Women’s age at first marriage and long-term economic empowerment in Egypt. World Development.

[b0625] Zan L.M., Rossier C., Studer M., Dao O., Guiella G. (2024). Contraceptive trajectories and women’s years of employment activity in low-income countries: Combining fictive cohorts, contraceptive calendar data and sequence analysis [Version 1]. VeriXiv.

[b0630] Zimmermann M., Sanz-Leon P., Stuart R., Driano E., O’Brien M. (2024). FPsim+: An agent based model of contraceptive use and empowerment [Version 1]. VeriXiv.

